# Case Report: Combined palatal augmentation and palatal lift prostheses for dysarthria and dysphagia in oculopharyngeal muscular dystrophy

**DOI:** 10.3389/fdmed.2026.1985199

**Published:** 2026-10-29

**Authors:** Ryo Tagaino, Naru Shiraishi, Jun Ohta, Nobuhiro Yoda, Hiroshi Egusa, Shigeto Koyama

**Affiliations:** 1 The Center for Dysphagia, Tohoku University Hospital, Sendai, Japan; 2 Maxillofacial Prosthetics Clinic, Tohoku University Hospital, Sendai, Japan; 3 Division of Advanced Prosthetic Dentistry, Tohoku University Graduate School of Dentistry, Sendai, Japan; 4 Department of Otolaryngology-Head and Neck Surgery, Tohoku University Graduate School of Medicine, Sendai, Japan; 5 Division of Molecular and Regenerative Prosthodontics, Tohoku University Graduate School of Dentistry, Sendai, Japan

**Keywords:** duplicate denture, dynamic impression, oculopharyngeal muscular dystrophy, palatal augmentation prosthesis (PAP), palatal lift prosthesis (PLP)

## Abstract

Oculopharyngeal muscular dystrophy (OPMD) is a rare progressive disorder characterized by severe dysphagia and dysarthria. Palatal lift prostheses (PLP) and palatal augmentation prostheses (PAP) are used to improve dysphagia and dysarthria; however, evidence for their combined use (c-PAP/PLP) in neuromuscular diseases is limited. We report a 68-year-old man with dysarthria and dysphagia due to OPMD who was initially managed with percutaneous endoscopic gastrostomy. Treatment with c-PAP/PLP improved dysarthria, hypernasality, and oral and pharyngeal function, resulting in reduced pharyngeal residue and absence of aspiration. This case highlights the potential value of compensatory prosthetic intervention in progressive neuromuscular disorders.

## 1 Introduction

A palatal lift prosthesis (PLP) elevates a weak or paralyzed soft palate to assist in velopharyngeal closure, thereby reducing hypernasality during speech and preventing nasal regurgitation during swallowing (1). A palatal augmentation prosthesis (PAP) enhances tongue–palate contact, facilitating bolus transit to the pharynx and improving articulation (2). However, few studies have examined the combined use of PAP and PLP (c-PAP/PLP) in patients with neuromuscular diseases (1, 2), particularly in those with oculopharyngeal muscular dystrophy (OPMD), a late-onset, intractable myopathy characterized by slowly progressive dysphagia (difficulty in swallowing), dysarthria (a motor speech disorder, which can affect articulation in speech), and impaired oral function (3).

This report describes the use of c-PAP/PLP to improve dysphagia and dysarthria in a patient with OPMD.

## 2 Case description

A 68-year-old man was admitted to Tohoku University Hospital with aspiration pneumonia and severe dysphagia and was referred to the Center for Dysphagia for evaluation of swallowing and speech difficulties. He had experienced progressive dysphagia for 6 years. Although he had been consuming a regular diet prior to the visit, he gradually became unable to eat. At the first visit, his body mass index was 15 (height: 167.3 cm; weight: 43 kg) (Table 1). Oral and swallowing functions were evaluated at the Center for Dysphagia, and he was diagnosed with OPMD by a neurologist. Clinical examination revealed hypernasality and nasal air leakage during vocalization. Videoendoscopic examination (VE) showed poor soft palate elevation, saliva pooling, vallecular residue, and a delayed swallowing reflex. Videofluorographic examination (VF) demonstrated poor masticatory movements, reduced tongue propulsion, velopharyngeal incompetence (VPI), and aspiration due to reduced bolus propulsion by the tongue and poor laryngeal elevation, accompanied by an ineffective reflexive cough after swallowing (Figure 1A) (4). Jaw, tongue, and lip mobility were preserved; however, maximal isometric tongue pressure was low (4.10 ± 0.17 kPa), measured using a tongue pressure measurement device (TPM-01; JMS, Hiroshima, Japan) (Table 1), and the tongue insufficiently contacted the palate. Following a multidisciplinary swallowing conference, the patient was instructed to fast [nothing by mouth; The International Dysphagia Diet Standardisation Initiative (IDDSI) Level 0] (5) and underwent percutaneous endoscopic gastrostomy (PEG) for nutritional support. To compensate for reduced tongue pressure and VPI, a c-PAP/PLP was planned.

**Table 1:** Clinical progress and functional evaluation of the patient.

Evaluation		First visit	With the PAP	With the c-PAP/PLP	At 3-year follow-up (post-prosthetic)	Post-cricopharyngeal myotomy
Body weight (kg)		43	55	52	54.6	51
Nutritional status		Fasting (NG tube)	Oral intake + PEG	Oral intake + PEG	Oral intake + PEG	Total oral intake (PEG removed)
VE assessment with jelly	Pharyngeal residue	Severe	Reduced, but present	Present	Present	None
VF assessment with jelly	Aspiration	Present	Occasional	None	None	None
	Oral phase	Poor bolus propulsion and crushing	Improved propulsion, but oral residue is still present.	Improved propulsion more	Improved propulsion more	
Speech intelligibility 1: Well-understood 2: Sometimes not understood 3: Understood, if subject familiar with topic 4: Sometimes understood 5: Not understood at all		2: Sometimes not understood	2: Sometimes not understood	1: Well-understood	1: Well-understood	1: Well-understood
Tongue pressure (kPa)		4.10 ± 0.17	4.73 ± 0.35	6.50 ± 0.17	4.93 ± 0.32	5.60 ± 0.17

**Figure 1:**
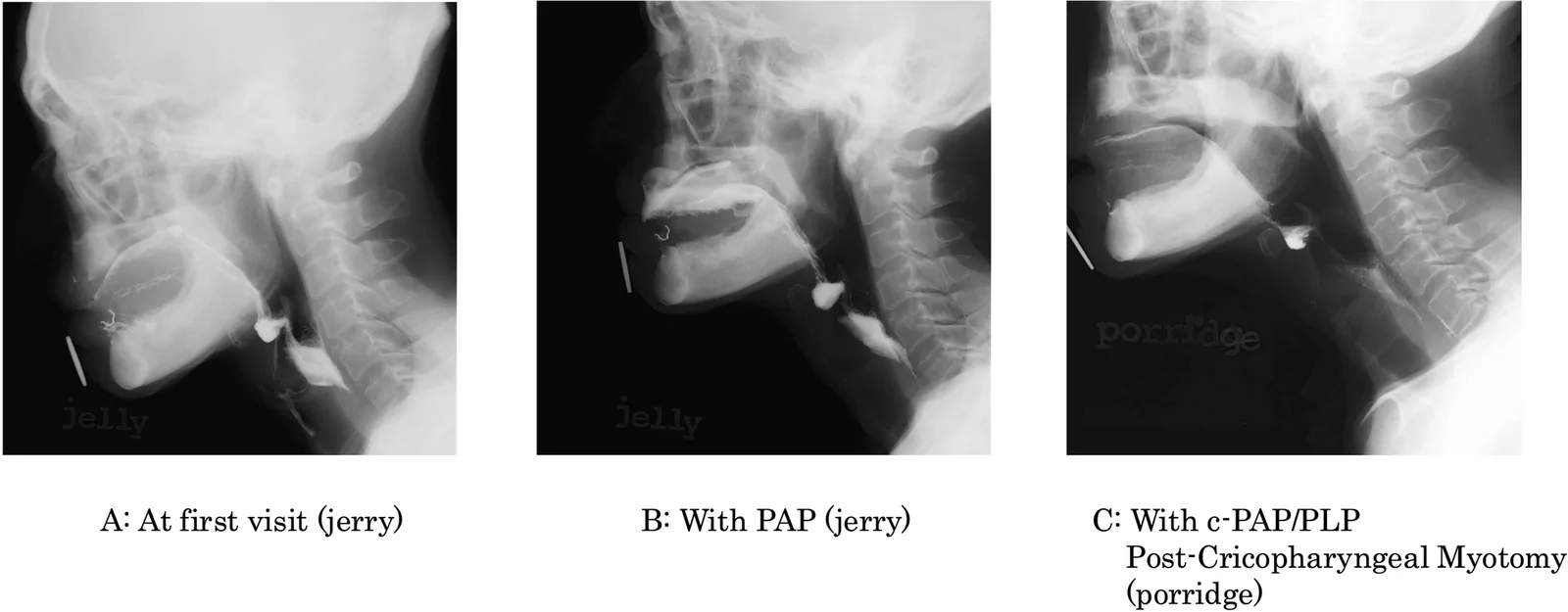
Videofluorography (VF) after swallowing jelly or porridge. All images represent the post-swallow phase. **(A)** At first visit (jelly). **(B)** With the PAP (jelly). **(C)** With the c-PAP/PLP, post-cricopharyngeal myotomy (porridge).

## 3 Diagnostic assessment and therapeutic intervention

The c-PAP/PLP was fabricated from a duplicate denture modified to incorporate both PAP and PLP components using a dynamic impression technique. Initially, the PAP portion was formed using a soft relining material (tissue conditioner II; Shofu, USA) applied to the denture palatal surface. The patient was instructed to perform pronunciation movements (/ta/, /ki/, and /ka/), swallow saliva, and press his tongue against the anterior palate. Necessary adjustments were made using a palatogram (Figures 1B, 2).

**Figure 2:**
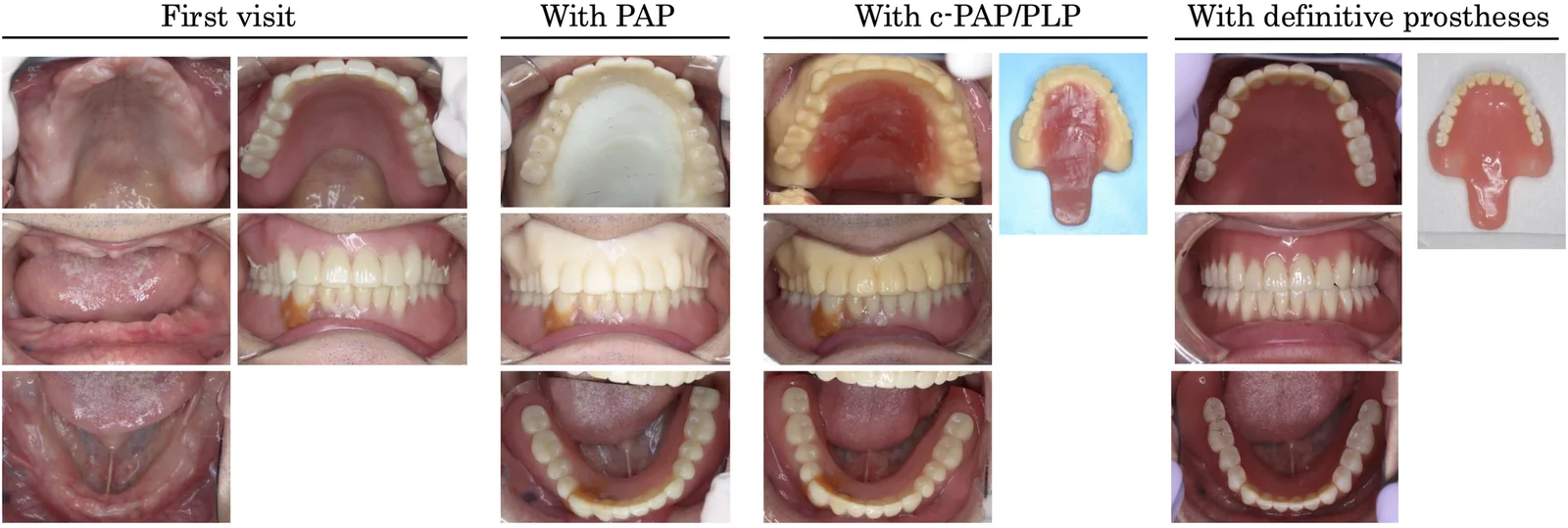
Intraoral views and dentures.

Subsequently, the PAP was converted to a PLP by adding self-curing resin (PROVINICE; Shofu, USA) to the posterior border to elevate the soft palate. The posterior border was extended approximately 5 mm per visit while monitoring the pharyngeal reflex and patient adaptation (Figure 2). Nasal air leakage and velopharyngeal closure were assessed using VE, and the length, lifting angle, and thickness of the PLP were adjusted accordingly (Figure 3). After confirming improvements in swallowing and speech function, definitive prostheses were fabricated using a dynamic impression technique (6).

**Figure 3:**
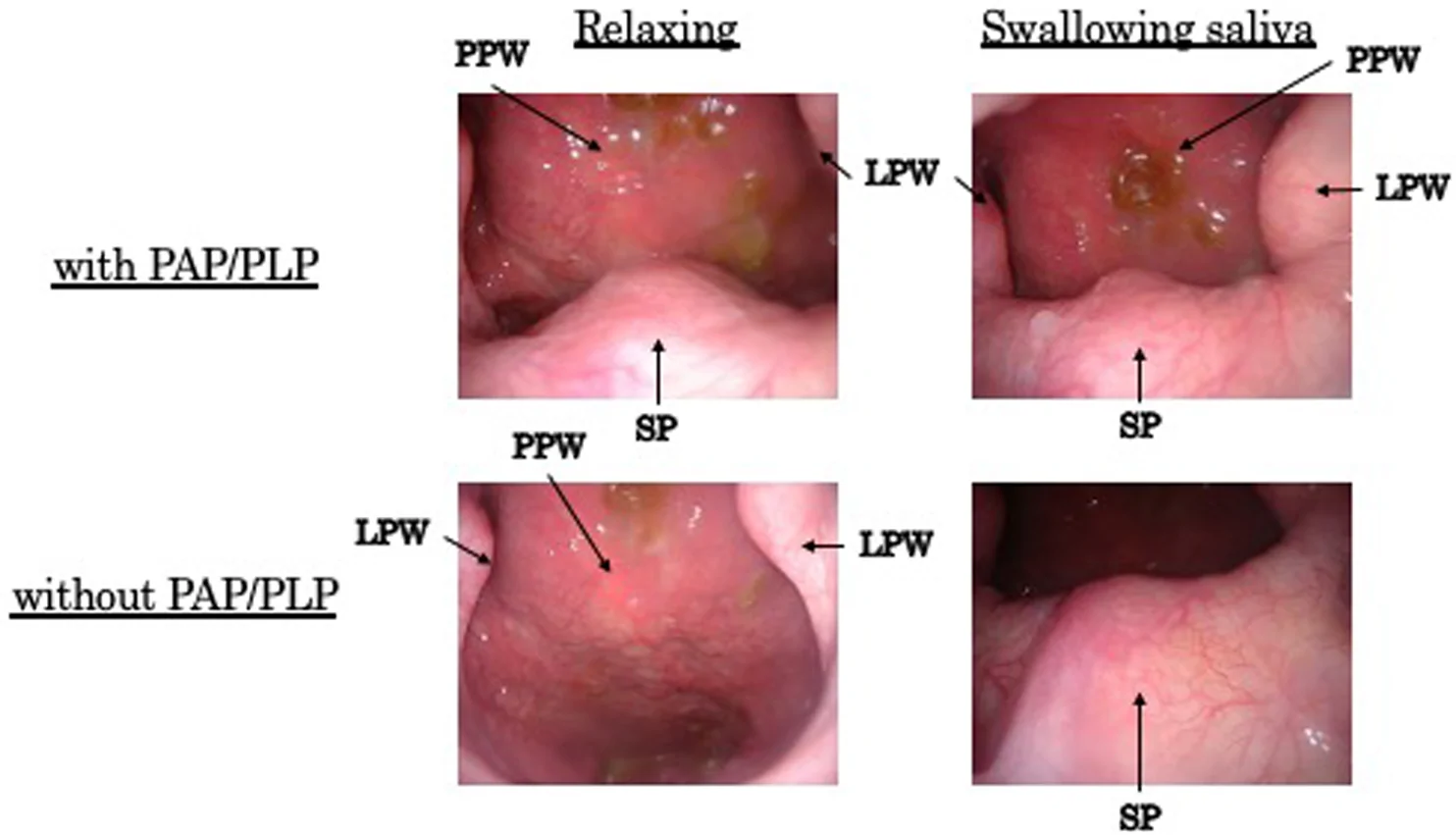
Videoendoscopic examination (VE) soft palate images during swallowing with and without the c-PAP/PLP. SP, soft palate; PPW, posterior pharyngeal wall; LPW, lateral pharyngeal wall.

## 4 Follow-up and outcomes

After delivery of the prostheses, oral and swallowing functions were re-evaluated. Although his maximal isometric tongue pressure remained relatively low (6.50 ± 0.17 kPa), the PAP enabled the tongue to contact the palate, improving the oral phase of swallowing. Concurrently, the PLP supported and elevated the soft palate and eliminated nasal regurgitation, resulting in improved swallowing and speech function, with reduced hypernasality and improved articulation. This prosthetic intervention yielded measurable improvements: speech intelligibility improved from Level 2 (“sometimes not understood”) to Level 1 (“well-understood”). Furthermore, VE and VF assessments confirmed that aspiration was successfully eliminated (Table 1). This functional recovery permitted the patient to transition from a fasting state to partial oral intake (IDDSI Level 4) alongside PEG nutrition, contributing to an initial weight gain. VE showed persistent saliva pooling in the valleculae and a delayed swallowing reflex. VF demonstrated slow but effective swallowing with reduction of pharyngeal residue and no aspiration. Tongue pressure, bolus transport to the pharynx, and pharyngeal residue gradually improved (Table 1) and the patient was eventually able to swallow thick liquids and soft food (IDDSI Level 4).

These functional improvements remained stable 3 years after delivery of the definitive prostheses. The patient's body weight increased from 43 to 54.6 kg. However, although the c-PAP/PLP improved the oral phase and velopharyngeal closure, the patient still exhibited pharyngeal residue due to underlying cricopharyngeal dysfunction. Over the 3-year follow-up, cricopharyngeal achalasia became the primary problem preventing safe swallowing, with VF demonstrating insufficient upper esophageal sphincter opening.

To address these dysfunctions, a cricopharyngeal myotomy was subsequently performed (Figure 1C). This surgical intervention enabled sufficient oral intake to meet total nutritional requirements. Following the recovery of swallowing function, the patient became independent of tube feeding, and the PEG tube was eventually removed. The patient's velopharyngeal closure function, which had become impaired due to the progression of OPMD, was assessed, and the shape of the palatal lift parts was modified in response to these functional changes.

## 5 Discussion

In this case, a multidisciplinary approach that included early nutritional support via PEG and a compensatory intervention using a c-PAP/PLP improved dysarthria and dysphagia associated with reduced tongue pressure and insufficient soft palate elevation through synergistic effects in a patient with OPMD.

This study has several limitations because it is a single case report. The progressive nature of OPMD and lack of a control intervention limit the generalizability of these findings. Furthermore, because the patient received a multidisciplinary approach, including nutritional support, prosthodontic rehabilitation, and surgical intervention (cricopharyngeal myotomy), the observed long-term functional improvements cannot be attributed solely to the c-PAP/PLP. Rather, the prostheses served as a crucial component in correcting the oral and velopharyngeal deficits, paving the way for the subsequent surgical resolution of the pharyngeal deficits.

This case demonstrates the clinical usefulness of a compensatory prosthetic rehabilitation for improving swallowing and speech function in progressive neuromuscular disorders.

## Data Availability

The raw data supporting the conclusions of this article will be made available by the authors, without undue reservation.
